# Supplementary material for: COZOID: contact zone identifier for visual analysis of protein-protein interactions
Source: BMC Bioinformatics. 2018 Apr 6;19:125. doi: 10.1186/s12859-018-2113-6 (PMC5889581; doi:10.1186/s12859-018-2113-6)
Supplement: Supplementary file 2 — Software build. Executable binary file of the software tool. (ZIP 118,784 kb) [file 12859_2018_2113_MOESM2_ESM.zip › cozoid/java/docs/beansbinding-1.2.1-doc.zip!/org/jdesktop/observablecollections/package-tree.html]

org.jdesktop.observablecollections Class Hierarchy (Beans Binding)


---


|  |  |  |
| --- | --- | --- |
| **Overview** \| **Package** \| Class \| Use \| **Tree** \| **Deprecated** \| **Index** \| **Help** | |  |
| **PREV**   **NEXT** | **FRAMES**    **NO FRAMES**     **All Classes** |  |


---


## Hierarchy For Package org.jdesktop.observablecollections

**Package Hierarchies:**: All Packages

---

## Class Hierarchy

- java.lang.Object
  - org.jdesktop.observablecollections.**ObservableCollections**- org.jdesktop.observablecollections.**ObservableCollections.ObservableListHelper**<E>

## Interface Hierarchy

- java.util.EventListener
  - org.jdesktop.observablecollections.**ObservableListListener**
  - java.lang.Iterable<T>
    - java.util.Collection<E>
      - java.util.List<E>
        - org.jdesktop.observablecollections.**ObservableList**<E>
    - java.util.Map<K,V>
      - org.jdesktop.observablecollections.**ObservableMap**<K,V>
      - org.jdesktop.observablecollections.**ObservableMapListener**

---


|  |  |  |
| --- | --- | --- |
| **Overview** \| **Package** \| Class \| Use \| **Tree** \| **Deprecated** \| **Index** \| **Help** | |  |
| **PREV**   **NEXT** | **FRAMES**    **NO FRAMES**     **All Classes** |  |


---
